# Encapsulation of bacteriophage cocktail into chitosan for the treatment of bacterial diarrhea

**DOI:** 10.1038/s41598-021-95132-1

**Published:** 2021-08-02

**Authors:** Golnar Rahimzadeh, Majid Saeedi, Mahmood Moosazadeh, Seyyed Mohammad Hassan Hashemi, Amirhossein Babaei, Mohammad Sadegh Rezai, Kosar Kamel, Kofi Asare-Addo, Ali Nokhodchi

**Affiliations:** 1 Pediatric Infectious Diseases Research Center, Communicable Diseases Institute, Mazandaran University of Medical Sciences, Sari, Iran; 2 Department of Pharmaceutics, Faculty of Pharmacy, Mazandaran University of Medical Sciences, Sari, Iran; 3 Gastrointestinal Cancer Research Center, Non-communicable Diseases Institute, Mazandaran University of Medical Sciences, Sari, Iran; 4 Student Research Committee, Faculty of Pharmacy, Mazandaran University of Medical Sciences, Sari, Iran; 5 Department of Animal Science, Sari Agriculture Science and Natural Resources University, Sari, Iran; 6 Department of Pharmacy, University of Huddersfield, Huddersfield, HD1 3DH UK; 7 Pharmaceutics Research Lab, School of Life Sciences, University of Sussex, Brighton, UK

**Keywords:** Nanobiotechnology, Nanoscale materials, Gastroenterology, Phage biology

## Abstract

The therapeutic effectiveness of a chitosan encapsulated bacteriophage cocktail as a smart biocontrol agent was evaluated in this study to be used as a preventative and treatment option for gastrointestinal infections. To evaluate the effect of the bacteriophage formulation on the treatment of gastrointestinal infection, rats were infected with *Salmonella enterica*, *Shigella flexneri*, and *Escherichia coli*. The rats were weighed and their stools cultured. The results showed that the group which had the chitosan encapsulated bacteriophage cocktail did not lose weight after 3 days and had significantly lower group weight changes. Weight loss was significant in the rats that had cefixime administered instead. Positive cultured stools were reduced after 4 days compared to 2 days in the treated group with the chitosan encapsulated bacteriophage cocktail. The chitosan encapsulated bacteriophage cocktail can therefore be effective in the treatment of gastrointestinal infections.

## Introduction

Diarrhea is one of the most common types of gastrointestinal infections and a leading cause of death worldwide. Viruses, bacteria (*Salmonella enterica*, *Shigella flexneri*, *Escherichia coli)*, and parasites cause diarrhea, which can be acute or chronic^1,2^. It is recommended that patients with diarrhea be provided with supportive measures such as proper nutrition, hydration and antibiotics in some cases. However, in the experimental treatment for diarrhea, the antibiotics used for treating *E. coli O157* causes hemolytic–uremic syndrome (HUS), which is a deadly disease^3,4^. Antibiotic-associated diarrhea can turn acute diarrhea into chronic diarrhea. Moreover, the inappropriate use of antibiotics in common infections such as diarrhea has contributed to antibiotic resistance throughout the world^5^. Alternative treatments such as bacteriophages are thus suggested^6,7^.

Bacteriophages solely target and kill bacteria. Unlike antibiotics, they do not affect the normal bacterial flora. They have no effect on eukaryotic cells and hence are non-toxic. Phages proliferate at the infection site and contrary to antibiotics do not have dose limits. Their proliferation is rapid and cost-effective^6–9^. In 1960, commercially produced of phage against diarrhea with *Shigella flexneri* and *E. coli* was reported in Tbilisi. Two types of phages were also used to treat children with acute diarrhea at the Dhaka Hospital in Bangladesh^10^.

Limited host range and the emergence of resistance to phages are among the challenges faced in mono-phage therapy. The use of two or more phages through phage cocktails increases the host range and reduces the likelihood of phage resistance^11^.

To use phage cocktails for treating gastrointestinal infections, a drug delivery system is required to protect phages from being destroyed and inactivated under the acidic conditions in the stomach. This ensures that they maintain their structure and lytic activity when entering the intestines. Chitosan and its associated compounds such as trimethylammonium chitosan are normal polyatomic polysaccharides that have been used in a range of applications and include N-acetyl glucosamine and glucosamine units^12^. Chitosan has been used for the delivery of drugs such as insulin via multiple administration routes as a carrier in polymeric nanoparticles^13^. Chemical functional groups of chitosan can also be modified to achieve specific goals such as ocular targeting^14^ thus rendering it a versatile polymer with applications in antimicrobial agents, wound dressing adhesives, and biosensors. More recently, polymeric nanoparticles have been considered as a unique and powerful therapeutic and diagnostic agent^15^. Chitosan is biocompatible, non-toxic and biodegradable^16^. Although the oral toxicity of chitosan is negligible^17^, this may be dependent on the purity, molecular mass, deacetylation degree, and method of administration.

This current research intended to study the construction of a chitosan encapsulated bacteriophage cocktail against bacteria causing diarrhea. This was investigated through the use of rats that were infected with *Salmonella enterica*, *Shigella flexneri*, and *Escherichia coli*. Formulation effects were also evaluated on the treatment of the diarrhea through weight loss and the culturing of the rat’s stools.

## Methods

### Materials

In the microbial tests, the chocolate agar and blood agar were purchased from Merck, Germany. The Kirby–Bauer technique was performed by using antibiotic discs and all discs including colistin (10 µg), ampicillin (10 µg), nitrofurantoin (300 µg), tetracycline (30 µg) meropenem (10 µg), cefotaxime (30 µg), gentamicin (10 µg), cefixime (30 µg) and ciprofloxacin (10 µg) were purchased from Padtanteb, Iran. Luria–Bertani (Quelab, USA), agarose (ACROS, Belgium), ammonium acetate (ACROS, Belgium) and uranyl acetate (ACROS, Belgium) were used for the isolation and characterization of the bacteriophage. For the preparation of the encapsulation of bacteriophage cocktail into chitosan, sodium tripolyphosphate (STPP), gelation, acetic acid and chitosan were obtained from Merck, Germany. *Salmonella enterica* ATCC No. 13076, *Shigella flexneri* ATCC No.12022 and *E. coli* ATCC No. 35218 were purchased from the Pasteur Institute, Iran.

### Preparation of bacteria strains

*Salmonella enterica* ATCC No. 13076, *Shigella flexneri* ATCC No.12022 and *E. coli* ATCC No. 35218 were cultured on the chocolate agar and blood agar and incubated at 37 °C for 24 h. The susceptibility of the above bacteria to the antibiotics was determined by the Kirby–Bauer test with the antibiotic discs namely ampicillin (10 µg), nitrofurantoin (300 µg), tetracycline (30 µg) meropenem (10 µg), cefotaxime (30 µg), gentamicin (10 µg), cefixime (30 µg) and ciprofloxacin (10 µg). According to The Clinical & Laboratory Standards Institute (CLSI) guideline, colistin minimum inhibitor concentration (MICs) was determined with macro dilution. Based on the CLSI guideline colistin MICs were: susceptible ≤ 2 and resistant > 2 mg/L)^18^.

### Isolation of bacteriophage

The sewage sample (200 mL) was collected from the sewage at the tertiary pediatric Bou Ali Sina Hospital in Sari, Mazandaran province, Iran. The sample was transported to the laboratory and stored at 4 °C. An equal volume of 2X LB broths (Quelab, USA) was added to the sewage sample. *Salmonella enterica* ATCC No. 13076, *Shigella flexneri* ATCC No.12022, and *E. coli* ATCC No. 35218 strains were separately cultured overnight then the samples were incubated for 24 h at 37 °C in a shaker incubator^8,9^. After 24 h the three samples were centrifuged at 11,000 × *g* for 15 min. The supernatants (bacteriophage) were filtrated through a 0.22-µm syringe filter under sterile conditions and room temperature (24 °C). The filtered samples were mixed (phage cocktail) and then stored at 4 °C till required for further analysis^8,9^.

### Double layer agar (DLA) assay and enrichment of phage cocktail

Sodium chloride-magnesium sulfate buffer (100 mmol/L NaCl, 8 mmol/L MgSO_4_, 2% gelatin, and 50 mmol/L Tris–HCl [pH 7.5]) was sterilized by using an autoclave. Then 900 μL of this sterilized solution was added to 10 sterile tubes (labelled tubes 1–10). Phage cocktail (100 μL) stored at 4 °C was added to tube no. 1 and vortexed. After vortexing, 100 μL from tube no. 1 was taken out and added to tube no. 2. This procedure was repeated in a systematic manner until tube no. 8. Tube no. 9 and tube no. 10 were selected and labelled as the positive and negative control, respectively. From each of the diluted phage cocktails, 200 µL was transferred to 200 µL of the *E. coli* (1.5 × 10^8^ CFU/ mL). The mixtures were added to the top agar (0.8% agar) followed by the addition of the top agar to the bottom agar (1% agar) then incubated overnight at 37 °C. Plaque-forming unit (PFU) was calculated per milliliter by determining the number of plaques × 10 × the inverse of the dilution factor^8,9^.

The formed plaques were inoculated in 100 mL Luria–Bertani (Quelab, USA) containing the *E.coli* (1.5 × 10^8^ CFU/mL) for 24 h at 37 °C in a shaker incubator. The sample was then centrifuged at 11,000 × *g* for 15 min. The supernatant was filtrated using a 0.22 µm syringe filter under sterile conditions and room temperature (24 °C). The above experiment was repeated three times^8,9^. The above experiment was performed for *Salmonella enterica* and *Shigella flexneri* separately.

### Transmission electron microscopy (TEM)

In order to prepare the phage for TEM, the phage cocktail was centrifuged at 25,000 × *g* for 60 min. The obtained phage cocktail was washed with 0.1 M neutral ammonium acetate. The phage cocktail was then deposited on a carbon-coated copper grid and stained using 2% uranyl acetate (pH 4–4.5). The phage cocktail was then observed on the Zeiss EM 900 TEM at 100 kV^8,9^.

### Determination of the host range of phage cocktail

To determine the lytic activity of the phage cocktail, the spot test was performed. The overnight cultured *Salmonella enterica*, *Shigella flexneri* and *E. coli* were inoculated in top agar and were poured into the bottom agar. A specified volume of the phage cocktail supernatant (50 µL) was poured over the solidified agar. The plates were incubated for 24 h at 37 °C. The formation of the inhibition zone was checked. The experiment was repeated three times^8,9^.

### Phage cocktails stability in vitro

The stability of the phage cocktail was investigated under various environmental conditions such as temperatures and pH. The phage cocktail was incubated at 4, 22, 37 and 50 °C for 60 min to determine thermal stability. The stability of the phage cocktail was determined at pH 3, 5, 7, 9, and 11 for 60 min. The lytic activity of the phage cocktail was detected by the double layer agar (DLA) assay which was described previously. The experiments were repeated three times^19^.

### Endotoxin removal strategy from phage cocktail supernatant

A phage supernatant was transferred to a sterile and free pyogenic bottle. To the phage supernatant 3% (v/v) Triton X-100 was added and incubated for 30 min at room temperature (25 °C) with shaking at 200 rpm. To remove the Triton X-100, 12% activated carbon was added, followed by incubation for 30 min at room temperature (25 °C) under shaking conditions (200 rpm). The solution was then centrifuged at 10.000 × *g* for 10 min. The produced supernatant was passed through a 0.45 μm membrane to remove the residual activated carbon. The purified phage solution was then stored overnight at 4 °C and the phage titer was determined on the following day^20^.

### Limulus amebocyte lysate (LAL) test

To evaluate the removal of endotoxin from the phage supernatant the LAL test was performed based on clotting response according to the protocol kit (ENDOSAFE, USA) with a potency of 0.06 EU/mL and 5 EU/mL endotoxin limit. *Escherichia coli* 055: B5 was used as a positive control. The samples were incubated in a water bath for 1 h at 37 °C^20^. The experiments were repeated three times.

### Chitosan nanoparticles (CS-NPs) preparation

CS-NPs were formulated with aqueous sodium tripolyphosphate (STPP) solution using the ion gelation method. In an aqueous solution of acetic acid (1%, w/v), 500 mg chitosan was dissolved to make a 5 mg/mL concentration. Under magnetic stirring (150 rpm) at room temperature (25 °C), the aqueous solution of STPP was applied dropwise to 100 mL of the chitosan solution. CS-NPs were spontaneously produced when STPP solution was introduced during the stirring. To acquire the phage loaded CS-NPs (phage-CS-NPs), 0.1 mL of the phage solution (63 × 10^10^ PFU/mL) was added to the chitosan solution. Different process variables (stirring rate and pH) and formulation factors (weight ratio of STPP and chitosan), influencing the properties of the nanoparticles, were investigated for their effect on the particle size and drug entrapment efficiency^21^.

### Entrapment efficiency (EE%)

To determine EE%, phage-CS-NPs (CS-NPs loaded with phage) were subjected to centrifugation at 19,980 × *g* for 30 min (SIGMA; 3-30 KS: Germany), followed by filtration of the supernatant (pore size 0.22 μm). The quantity of phage in the filtered solution (unentrapped or free drug) was then calculated using equation) through DLA assay^21^.

1 $$EE\% = \frac{Total\;amount\;of\;drug - amount\;of\;unentrapped\;drug}{{Total\;amount\;of\;drug}} \times 100$$

### In vitro drug release study

The in vitro drug release experiment for the phage from simple solution and phage loaded nanoparticles (CS-NPs) were carried according to the protocol described by Chen et al. (2009) with some alteration^21^. The in vitro release of phage solution and optimized CS-NPs were evaluated in pH 1.2 and pH 6.8 to mimic the gastrointestinal pH condition. The release experiment was assessed by the membrane dialysis technique at 37 °C temperature. Briefly, phage solution and phage-loaded CS-NPs were placed in a dialysis bag (MWCO 12 kDa) and suspended in 900 mL pH of 1.2 for up to 3 h. The dissolution medium was maintained at 37 ± 0.5 °C in the beaker with a stirring rate of 100 rpm. Samples (1 mL) were collected at pre-determined time points of 0.5, 1, 2 and 3 h at pH 1.2 and immediately refilled with a fresh (1 mL) medium. The pH 1.2 medium was then replaced by pH 6.8 and the analysis was continued for a further 3 h. Samples (1 mL) were collected at the same pre-determined time points and refilled with the corresponding fresh (1 mL pH 6.8) medium. The concentration of phage was then calculated using the DLA assay^21^.

### Characterization of CS-NPs

Dynamic light scattering (DLS) through a Zetasizer Nano ZS device (Malvern Instruments Worcestershire: UK) with an angle of 90° at 25 °C was used to determine the diameter and polydispersity index (PDI) and the size of the produced nanoparticles^22^. Laser Doppler electrophoresis was used to measure the zeta potential of the nanoparticles.

### Experimental model

**Figure 1:**
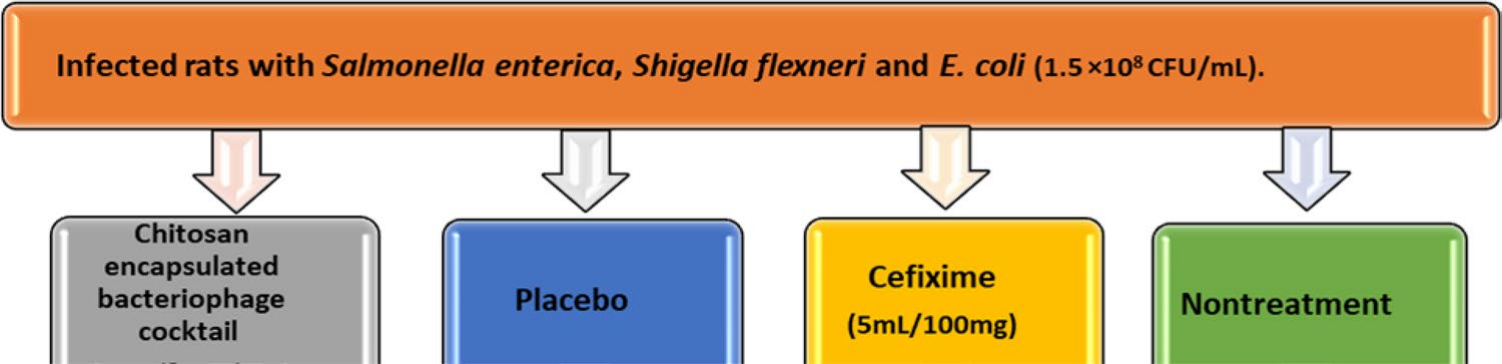
The rats were infected with 0.5 mL the bacteria (1.5 × 10^8^ CFU/ mL). They were divided into 4 main groups (N = 8).

Ninety-six Wistar female rats (8 weeks old, 180 ± 200 g of weight) were caged under controlled conditions of light, room temperature, and humidity for a week before the study. This study was approved by the ethical committee of Mazandaran University of Medical Sciences, Sari, Iran (IR. MAZUMS.REC.1399.1333). Rats were weighed on the day of the beginning of the experiment. They were deprived of food for 24 h before beginning the experiment. Gastrointestinal infection was induced by the use of 0.5 mL of *Salmonella enterica*, *Shigella flexneri* and *E. coli* suspension (1.5 × 10^8^ CFU/mL). After 48 h the rats were divided into four main groups with 8 rats each in each group (Figure 1). Here, 0.5 mL of the phage cocktail (10^10^ PFU /mL) was orally gavaged daily. The same volume (0.5 mL) was used in the group taking chitosan nanoparticles. In the positive control group, cefixime was gavaged as 100/5 mg/kg in rats daily, and the final group which did not take any treatment.

To evaluate the effect of the formulation on the treatment of gastrointestinal infection, rats were weighed on the 2nd, 4th, 6th and 8th day. The stool samples were cultured and counted on blood agar, MacConkey agar, and selective medium as Xylose Lysine Deoxycholate agar (XLD), Salmonella Shigella agar (SS) and Hecktoen enteric agar on the same days (2nd, 4th, 6th and 8th day). The positive cultures were determined by deferential microbial tests as catalase test, oxidase test, TSI test, Simmons' citrate agar test and Methyl Red / Voges-Proskauer (MR*/*VP) test^23^. The study is reported in accordance with ARRIVE guidelines. Meanwhile, all methods were carried out in accordance with relevant guidelines and regulations.

### Statistical analysis

The data were analyzed using the SPSS 23 software. The Kruskal–Wallis test was used. A value of *p* < 0.05 was considered statistically significant.

## Results

### Preparation of bacteria strains

The Kirby–Bauer test showed that the *E. coli* was sensitive to nitrofurantoin, cefotaxime and cefixime but resistant to ampicillin, tetracycline, meropenem, gentamicin and ciprofloxacin. *Salmonella enterica* and *Shigella flexneri* were sensitive to cefixime and resistant to ampicillin, tetracycline, meropenem, gentamicin, ciprofloxacin, nitrofurantoin and cefotaxime. Colistin MICs of *E. coli*, *Salmonella enterica* and *Shigella flexneri* were determined to be 0.5 (mg/L) , 0. 5 (mg/L) and 0.25 (mg/L), respectively. *Escherichia coli*, *Salmonella enterica* and *Shigella flexneri* were sensitive to colistin.

### Characterization of bacteriophage

**Figure 2:**
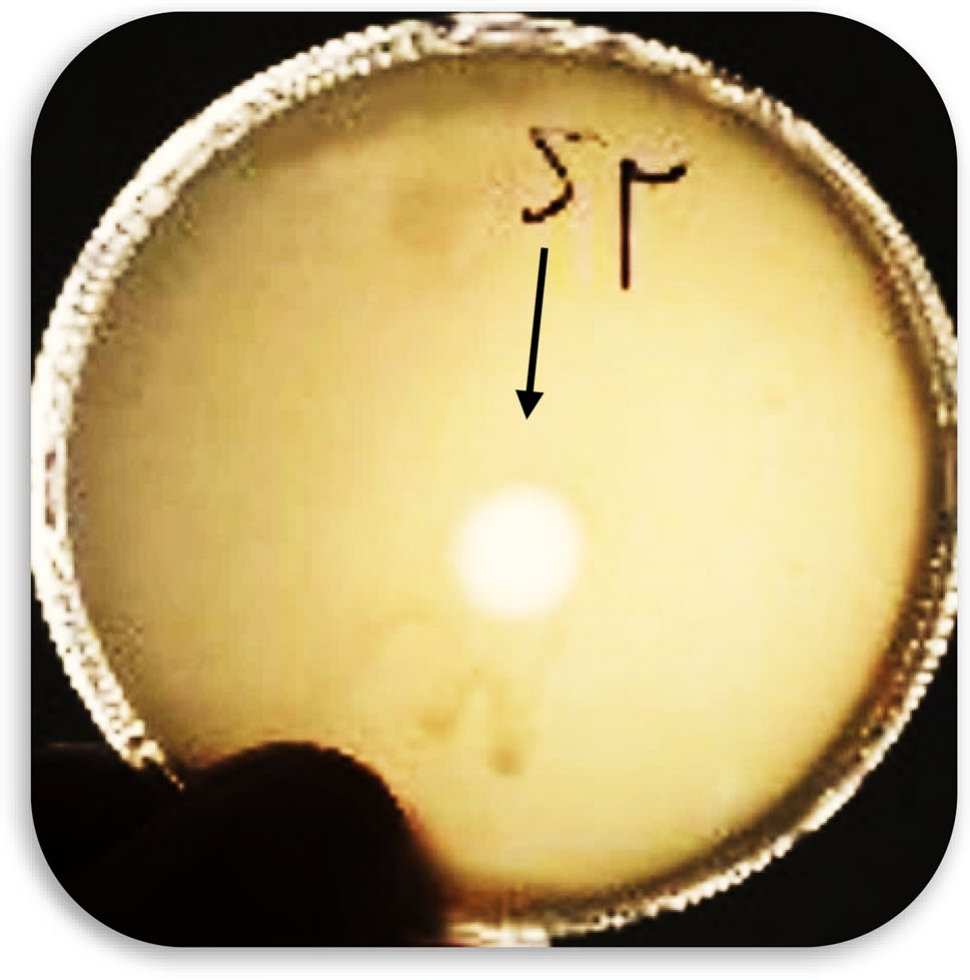
The spot test which shows formation of the inhibition zone and lytic activity of phage cocktail.

**Figure 3:**
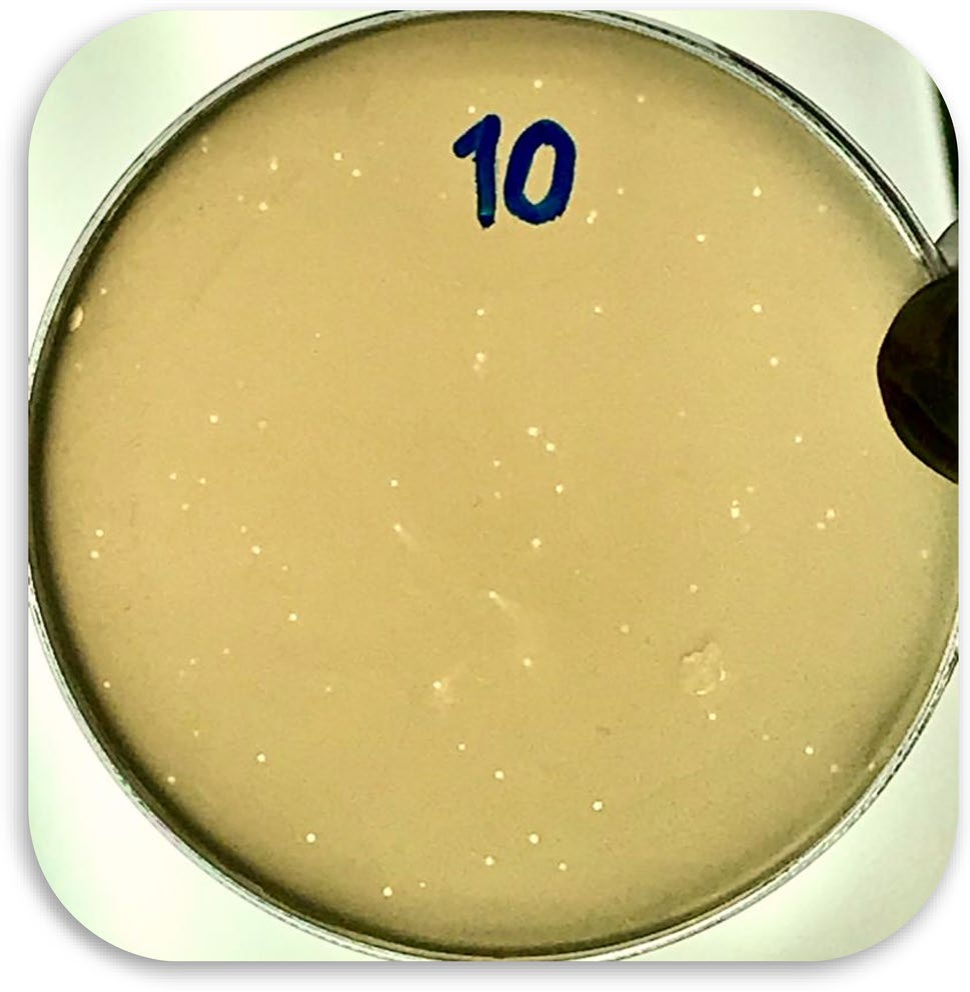
The titer phage cocktail (63 × 10^10^ PFU/mL) after three enrichments.

**Figure 4:**
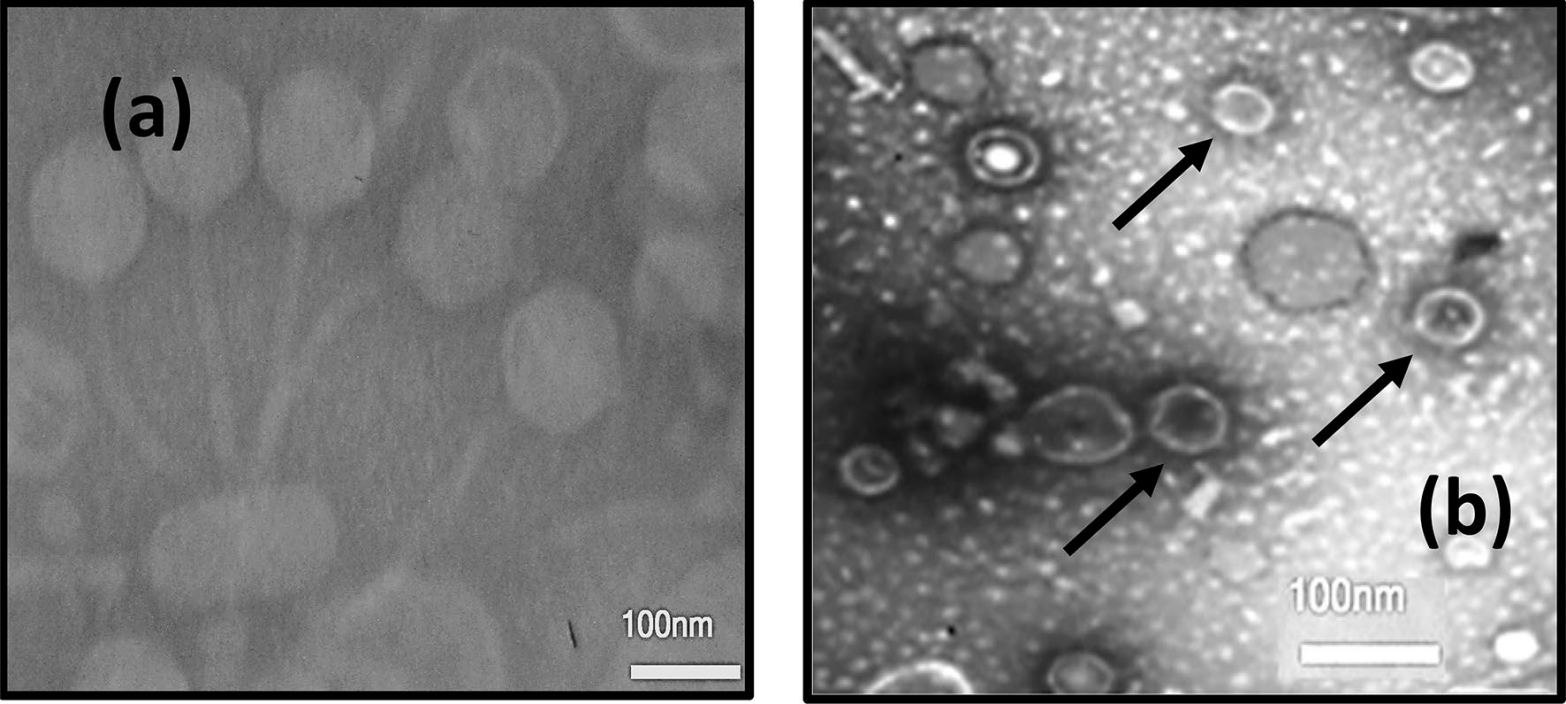
(**a**) Electron micrographs of phage belonging to the *Siphoviridae* family. (**b**) The *Cystoviridae* family. Note: Stained with 2% uranyl acetate (pH = 4–4.5). Voltage was 100 kV and the scale bar is 100 nm.

In the present study, the phages were isolated from a sewage sample. Figure 2 shows the lytic activity of the phage cocktail determined by the formation inhibition zone in the spot test. The titer phage cocktail was calculated (63 × 10^10^ PFU/mL) after four enrichments (Fig. 3). The morphology of the phage cocktail is depicted as (Fig. 4) by TEM. The two phages were observed in this image including the *Siphoviridae* with an icosahedral head (50–80 nm) and a long non-contractile tail (400 nm), and *Cystoviridae* with a spherical shape (80–100 nm) and a lipid membrane around the capsomere (Fig. 4).

### Effects of temperature and pH on the stability of phage cocktail

**Figure 5:**
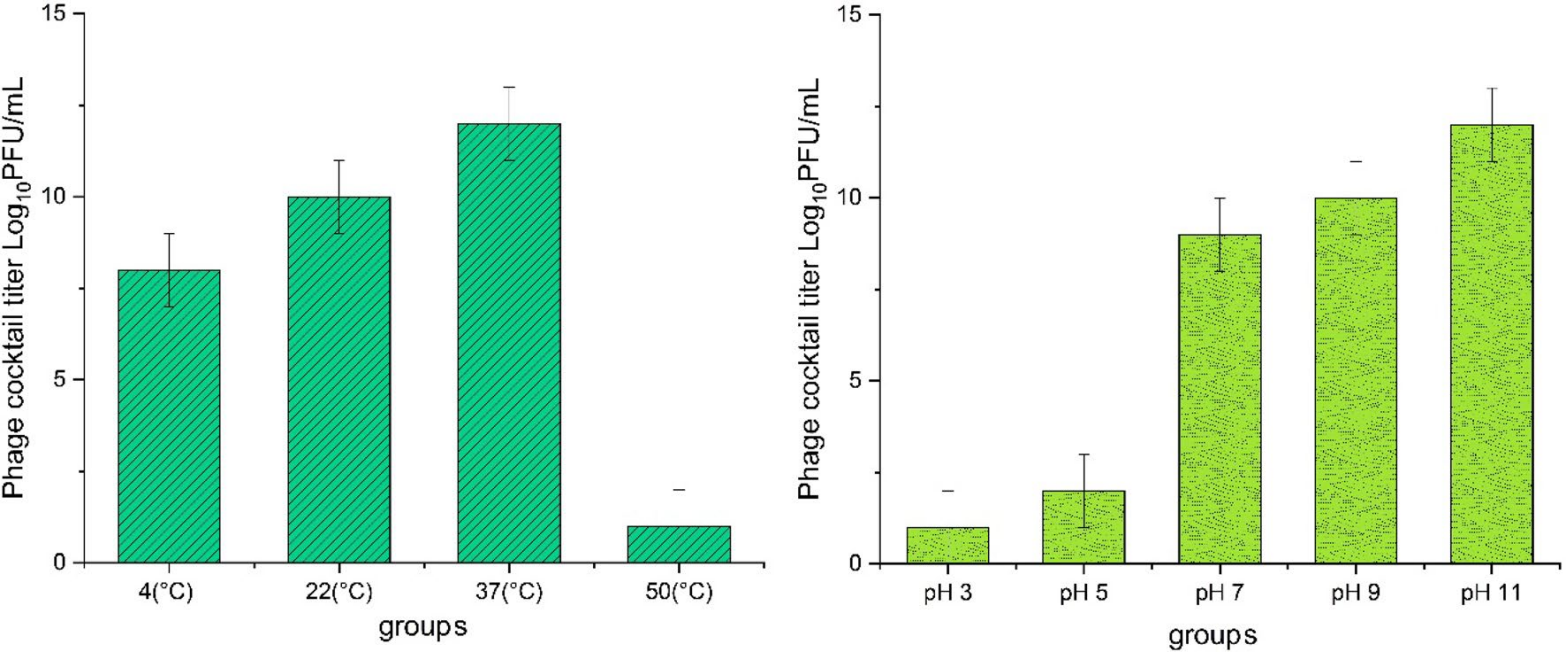
The titer of phage cocktail in various temperatures and pHs.

The viability of the behavior of the phages was considered in various conditions of temperature and pH. The phage cocktail showed the highest titer at 37 °C, but no active phages were found at 50 °C. The highest and lowest titer of phage cocktail was at pH 11 and 3, respectively (Fig. 5).

### Limulus amebocyte lysate (LAL) test

In this study, the endotoxin was removed from the phage cocktail using a two-phase extraction with Triton X-100 and activated charcoal. After detoxification, a reduction in the titer and validity of phage was not observed. The result of the LAL test to evaluate the removal of endotoxin in the phage supernatant with the potency of 0.06 EU/mL and 5 EU/mL endotoxin limit, did not form cloth after 1 h at 37 °C. In the positive control, the cloth was observed after 1 h at 37 °C.

### Chitosan nanoparticles (CS-NPs) preparation

The chitosan nanoparticles were prepared using the ionic gelation technique and the particles were optimized by employing various ratios of chitosan: STPP in terms of the entrapment efficiency and particle size (Table 1). The size of nanoparticles was found to increase from an unstable formulation (where no particles could be detected) to 298.2 ± 2.45 nm on increasing the ratio of chitosan: STPP from 1:1 to 4:1. The chitosan: STPP ratio at 4:1 was found to be optimal. Table 1 shows that the stability of nanoparticle suspensions diminished when the cross-linking agent concentrations increase. In addition, the formulation was less stable when the concentrations of both polymer and STPP were high.

**Table 1:** Optimization parameters for CS-NPs.

Formulation	Chitosan (mg/mL)	STPP (mg/mL)	Chi:STPP	Appearance	Size (nm)	PDI	EE (%)
F1	5	10	1:1	Immediately precipitated	–	–	–
F2	5	10	2:1	Immediately precipitated	–	–	–
F3	5	10	3:1	Immediately precipitated	–	–	–
F4	5	10	4:1	No precipitate	298.2 ± 2.4	0.431 ± 0.02	80 ± 5.5

### Characterization of CS-NPs

**Figure 6:**
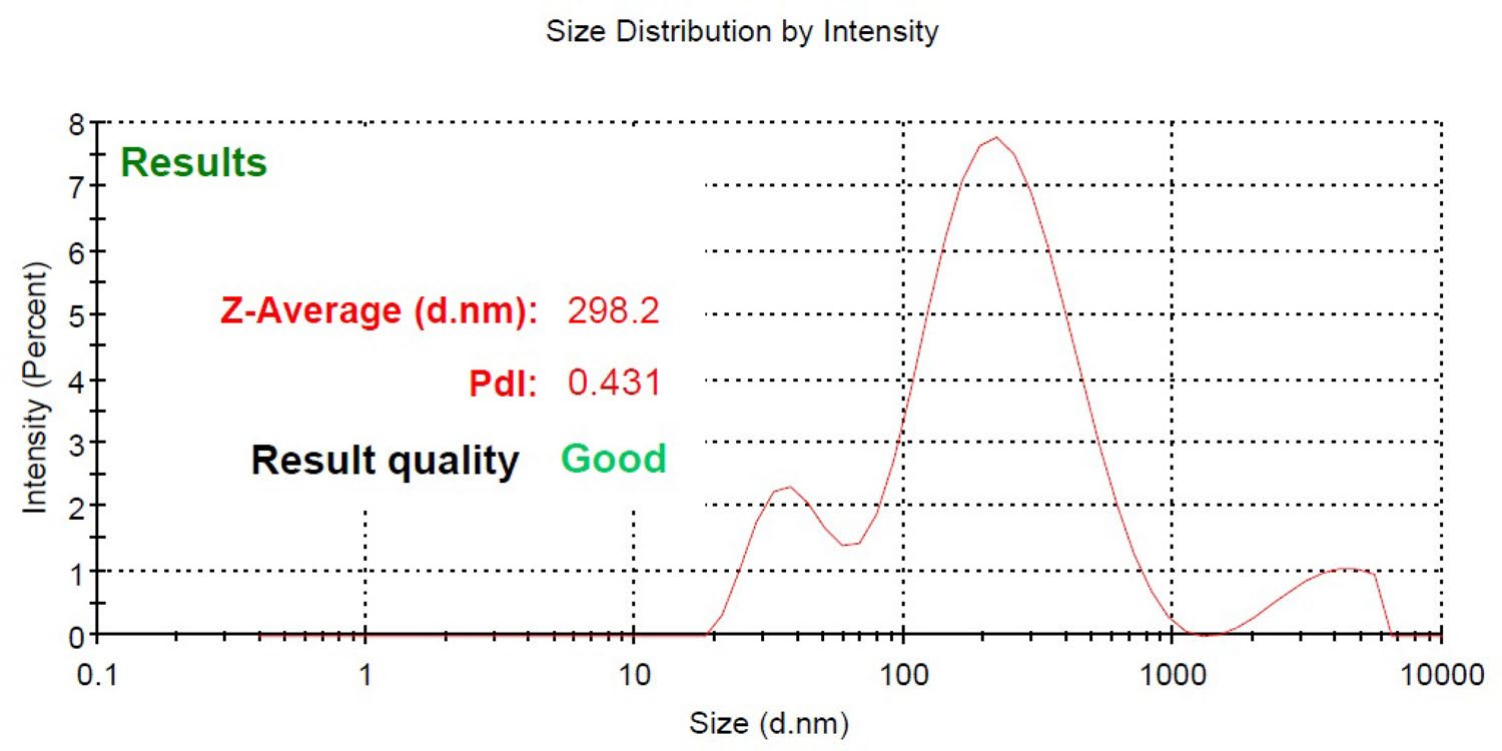
Dynamic light scattering results of phage chitosan nanoparticles (size distribution by the intensity and polydispersity index value).

Chitosan: STPP ratio 4:1 was found to be optimal because it had an acceptable size range (298.2 ± 2.45 nm) with a low polydispersity index (0.431 ± 0.02) and also improved drug entrapment efficiency (80 ± 5.5%) (Fig. 6).

### In vitro drug release study

**Figure 7:**
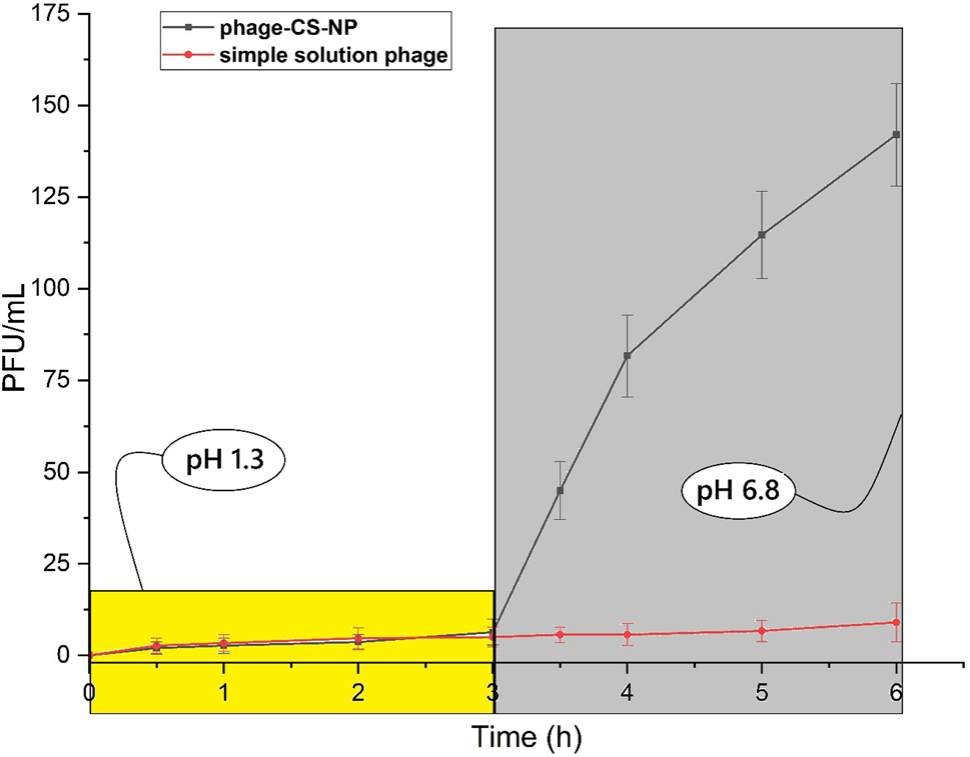
In vitro drug release study of phage-CS-NPs and phage solution in different pHs. Values represent mean ± S.D (n = 3).

In vitro drug release showed that in contrast to the simple solution phage which was not stable at acidic pH, phage-CS-NP is stable at pH 1.3. It may seem that the CS-NP protects the phage from the acidic condition and makes it more viable (Fig. 7).

### Experimental model

**Figure 8:**
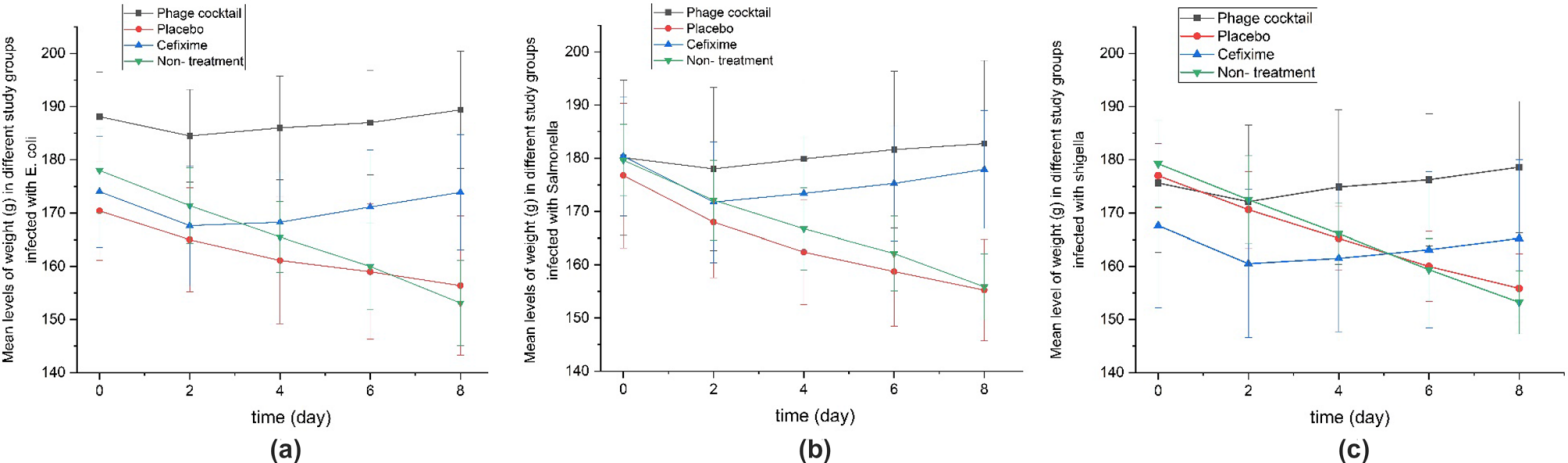
Weight changes in different treatment groups: (**a**) infected rats with *E. coli*, (**b**) infected rats with *Salmonella enterica*, (**c**) infected rats with *Shigella flexneri*.

In the experimental method in the group treated with chitosan encapsulated bacteriophage cocktail (phage-CS-NP), no weight loss was observed after 3 days. The weight change in this group was the lowest (*p* < 0.001). A normal weight gain was observed in this group which indicates the effectiveness of the phage cocktail in the treatment of diarrhea. In the treated group with cefixime, weight loss was significant compared to the chitosan encapsulated bacteriophage cocktail group (*p* < 0.05). The weight loss for rats in the non-treated and chitosan nanoparticles groups was significantly higher than the other two groups (groups treated with phage-CS-NP and cefixime) (*p* < 0.001) (Fig. 8a–c).

**Figure 9:**
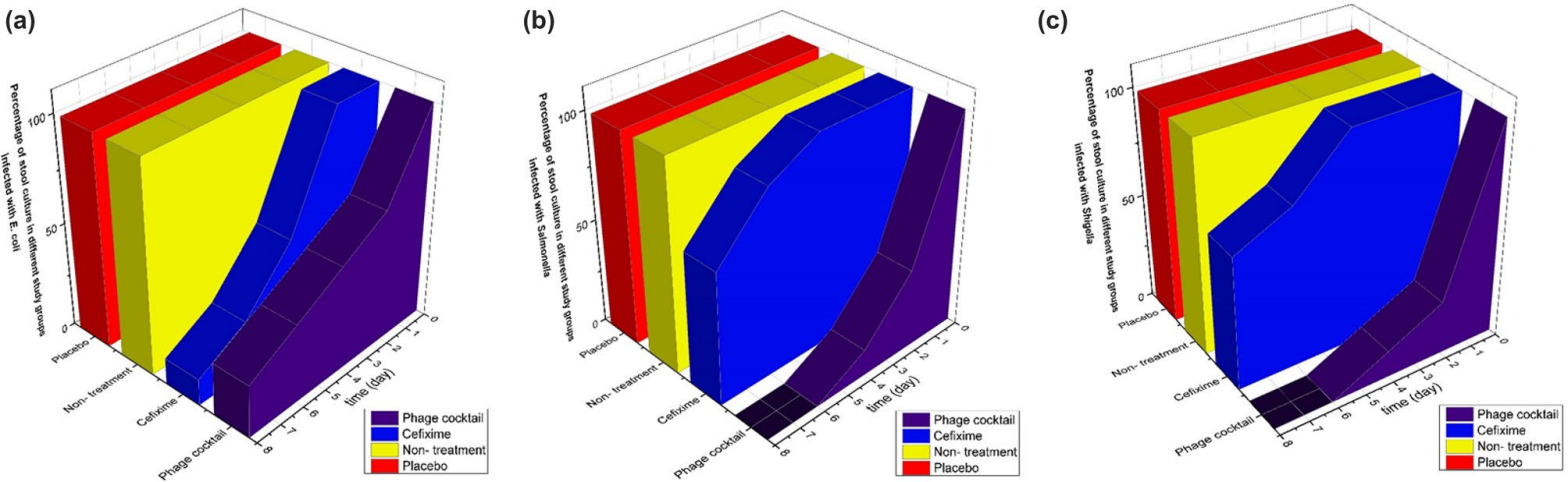
Percentage positive cultures in different treatment groups: (**a**) infected rats with *E. coli*, (**b**) infected rats with *Salmonella enterica*, (**c**) infected rats with *Shigella flexner*.

Based on the results of stool cultures, the number of positive cultures in the chitosan encapsulated bacteriophage cocktail group was reduced after 4 days compared to 2 days. In the cefixime group, *Salmonella enterica* and *Shigella flexneri* cultures were positive after 8 days. All cultures in the chitosan nanoparticles and non-treatment groups were positive on all days (Fig. 9a–c).

## Discussion

With emerging antibiotic resistance, infectious diseases are increasing and the need for an alternative option is essential^3–5^. Numerous studies have evaluated the potential of phage therapy for the treatment of gastrointestinal infections^6,11,24^.

Bacteriophages are found in the environment including soil, sewage, and animal wastes^24,25^. In the present study, the phage cocktail was isolated from sewage. The spot test determined the host range and lytic activity of the phage cocktail. In phage therapy lytic phages are useful. The lysogenic phages encode bacterial virulence factor including bacterial toxins and transfer resistance antibiotic genes^24^. No side effects on the normal flora of the gastrointestinal tract was therefore expected. Similar to our study Yang et al. (2018) isolated *Podoviridae* against *Salmonella enterica* from sewage samples with titer 10^10^ PFU/mL^19^.

Chibani-Chennouf et al. (2004), isolated four *Myoviridae* phage cocktails against Enterotoxigenic *E. coli* (ETEC) and Enter Pathogenic *E. coli* (EPEC) from environmental water and stool samples of pediatric patients with diarrhea^26^. Based on the results obtained in the current study, the phage cocktail can be used for *E. coli* (ETEC) and *E. coli* (EPEC).

Tamhankar et al. (2019) also isolated *Podoviridae* and *Myoviridae* phage cocktail against *E. coli*, *Enterobacter* species from sewage water treatment plants^27^.

In present study the highest titer of phage cocktail showed at 37 °C. The highest and lowest titer of phage cocktail was at pH 11 and 3, respectively. In a study carried out by Yang et al. (2018), it was shown that nearly 80% of the phage was viable after 24 h incubation at 50 °C with no active phages found after 30 min at 70 °C^19^. Tamhankar et al. (2019) reported the lytic activity of three phage cocktails to 55 °C and a reduction in their activity^27^.

The differences reported in various publications could be due to differences in bacterial strains, type of phages and sampling methods. Similar to this study, Yang et al. (2018) reported SFPH2 phage to exhibit high stability within pH ranges of 3–11after 24 h, however, it showed no titer at pH 2 and 13^19^. Tamhankar et al. (2019) also reported three phage cocktails to be inactivated at pH ≤ 3 and ≥ 12^27^.

The endotoxins were removed from the phage cocktail using a two-phase extraction with Triton X-100 and activated charcoal. According to Petsch and Anspach, an endotoxin removal efficacy of up to 93.21% and 99.96% could be observed for the φET and φCET phage samples, respectively^20^.

Thechitosan nanoparticles (CS-NPs) were prepared using the ionic gelation technique. Kaikabo et al. used a simple coacervation process to manufacture chitosan nanoparticles (CS-NP) and chitosan-phage-loaded nanoparticles^28^. The goal was to achieve a successful defence of bacteriophage against gastrointestinal digestive enzymes in the intestinal tract of a chicken. The average diameter for the CS-NP and phage-CS-NP were 188 ± 7.4 and 176 ± 3.2 nm, respectively^28^.

In the present analysis, three formulations were not stable during the preparation or after preparation. The stability of nanoparticle suspensions diminished when the cross-linking agent concentrations increased. In addition, the formulation was less stable when the concentrations of both polymer and STPP were high. In conclusion, the agglomeration of nanoparticles is encouraged by a decrease in the zeta potential as a result of increasing the quantity of STPP. This impact can be minimized if the particles are very dense, which is often caused by greater quantities of STPP. On the other hand, an excessive amount of cross-linker could facilitate accumulation due to the higher likelihood of inter-particle cross-linking.

One essential reason for the encapsulation of phage into CS-NPs was that the phage could not be destroyed by enzymes and stomach acid when administered orally and also was effectively transported to the site of action. Oral ingestion of the phage leads to a reduction in its viability which causes it to be inactivated. The findings of this analysis showed that the encapsulation of phage in CS-NP as a carrier can stop the phage from being enzymatically degraded relative to the simple solution phage that is degraded in vitro at pH 1.3. The pattern of release was probably due to the swelling of the matrix of chitosan, which promotes the protonation of their amino groups in the acidic condition contributing to the disintegration and gradual diffusion of the phage from the nano-polymers matrix. These findings are in agreement with the study carried out by Kaikabo et al.^28^ This demonstrated the ability of CS-NPs to prevent phage from degradation by the enzyme pepsin in vitro experiment. As proven in gel electrophoresis, phage encapsulated in chitosan nanoparticles are preserved from the degradation by acidic conditions and enzymes whilst naked bacteriophages are degraded under these conditions^28^.

In the current study, rats were placed into various groups. In the group treated with chitosan encapsulated bacteriophage cocktail, no weight loss was observed after 3 days which indicates the effectiveness of the phage cocktail in the treatment of diarrhea. The results showed that the weight loss in rats in the non-treated and chitosan nanoparticles alone groups were significantly higher than the other two groups (groups treated with phage-CS-NP and cefixime) (*p* < 0.001). Chitosan nanoparticles alone did not show antimicrobial activity against *Salmonella enterica* ATCC No. 13076, *Shigella flexneri* ATCC No.12022 and *E. coli* ATCC No. 35218 strains. Based on the results of stool cultures in chitosan nanoparticles alone, all cultures in this group and non-treatment groups were positive on all days*.*

In the present study, the phage cocktail was gavaged daily. Tanji et al. (2005) demonstrated that a single administration of a phage cocktail was not effective in reducing the *E. coli* concentration in the gastrointestinal tract. Daily administration of a high titer of phage cocktail was found to be effective in reducing the *E. coli* from the gastrointestinal tract^25^.

Vahedi et al. (2018) used *Myoviridae* bacteriophage against *E. coli* (EPEC). The single dose of the bacteriophage (2 × 10^9^ PFU/ mL) was able to control the infection after 10 days^29^. Nikkhahi et al. (2016) also used a single phage (2 × 10^9^ PFU/ mL) against *Salmonella enterica*. Their results showed that the bacteriophage was able to control diarrhea after 7 days^30^. In this study, we report the phage cocktail to have the highest lytic activity and control the infection after 3 days.

In previous studies, phage alone has been used in the treatment of diarrhea, but as the acidic conditions of stomach can inactivate the phage, this increased the duration of treatment^25–27^. In this study, phage was encapsulated into CS-NPs to protect it from the acidic conditions of the stomach. It was therefore present with the highest titer, which in turn, decreased the duration of recovery of diarrhea. In a study carried out by Rebenaque et al. (2021), phage FGS011 was encapsulated in two different pH-responsive formulations with Eudragit L100, and Eudragit S100. The pH of the proventriculus /gizzard in young chicks is however not sufficiently acidic to cause differential phage titre reductions, thereby allowing free phage survival in vivo^31^. The environmental conditions can therefore be effective on the lytic activity of phage.

Maura et al. (2012) reported that the phage cocktail (containing 3 phages: CLB_P1, CLB_P2, and CLB_P3) reduced the concentration of *E. coli* 55989Str (1 × 10^6^ CFU/g) in the ileal on day 4. But the concentration of bacteriophage was less efficient in the large intestine and feces than in the ileum. Local conditions (pH, nutrients, and electrolytes) in some spatial niches within gut parts may also affect bacteriophage permissively^32^. In this study, the phage was protected from the harsh local conditions experienced in the stomach as the phage cocktail was encapsulated in CS-NPs as a carrier. The chitosan may be processed in different nanomaterial forms that have enormous potentials to be applied as drug delivery systems, tissue engineering scaffolds, wound dressing adhesives, antimicrobial agents, and biosensors. Chitosan has been shown to be non-toxic, biocompatible and biodegradable^12^.

Denou et al. reported the effect of T4 phages in *E. coli* diarrhea in infected female mice which orally received phage (10^9^ PFU/ mL). Similar to our results, their weight normally increased, and animal functions such as eating, drinking and mobility were quite normal. These data thus confirm the effectiveness of the phage in eliminating the infection without an adverse effect on animal growth^33^.

## Conclusions

In this study, chitosan encapsulated bacteriophage cocktail treatment of diarrhea was successfully developed. The effect of the developed smart formulation for the treatment of diarrhea was compared to cefixime. The results proved that the encapsulated technique can be used to protect bacteriophage cocktail from the harsh conditions of the stomach such as the low pH of the stomach.

## Data Availability

Data is available on request.
